# Evaluation of Inflammatory Markers in Patients Undergoing a Short-Term Aerobic Exercise Program while Hospitalized due to Acute Exacerbation of COPD

**DOI:** 10.1155/2020/6492720

**Published:** 2020-04-28

**Authors:** Caroline Knaut, Carolina Bonfanti Mesquita, Victor Zuniga Dourado, Irma de Godoy, Suzana E. Tanni

**Affiliations:** UNESP-Univ Estadual Paulista, Campus de Botucatu, Department of Internal Medicine, Pneumology Area, SP, Brazil

## Abstract

**Introduction:**

Acute exacerbation is an important factor for a worse prognosis in patients with chronic obstructive pulmonary disease (COPD). It promotes the increase of the inflammatory process and worsens quality of life, lung function, and muscle weakness. It is believed that physical exercise performed during the exacerbation breaks the vicious cycle of systemic manifestations without an increase in the inflammatory process.

**Objective:**

To evaluate the influence of short-term aerobic physical exercise during hospitalization on inflammatory markers. *Patients and Methods*. 26 patients were evaluated (69.2% female, FEV 137.5 ± 12.9%, and age 68.4 ± 11.6 years) 24 hours after hospitalization for smoking history, Charlson index, quality of life, systemic inflammatory markers, and body composition. After 48 hours of hospitalization, all patients underwent a 6-minute walk test (6MWT) and a new spirometry test, and BODE index was calculated. After 72 hours of hospitalization, patients in the intervention group underwent aerobic exercise on a treadmill for 15 minutes twice daily; before and after the aerobic exercise, blood samples were collected for evaluation of inflammatory markers. Finally, a month after hospital discharge, all patients were reevaluated according to systemic inflammatory markers, quality of life, body composition, spirometry, 6MWT, and BODE index.

**Results:**

Patients of both groups did not differ in severity of disease and general characteristics. The intervention group did not show worsening in the inflammatory process after aerobic activity: TNF-*α* from 1.19 (0 99–1.71) to 1.21 (0.77–1.53) (*p* = 0.58), IL-6 from 2.41 (2.02–0.58) to 2.66 (1.69–0.48) (*p* = 0.21), and CRP from 3.88 (2.26–8.04) to 4.07 (2.65–13.3) (*p* = 0.56). There was a negative correlation between the IL-6 marker and the 6MWT; that is, with the reduction in inflammatory levels, there was an improvement in exercise capacity one month after hospital discharge.

**Conclusion:**

The present study showed that the aerobic physical activity initiated during hospitalization in patients with exacerbated COPD did not worsen the inflammatory process.

## 1. Introduction

International guidelines define chronic obstructive pulmonary disease (COPD) as a disease with airflow limitation, including abnormalities of the airway and alveolar abnormalities caused by exposure to noxious particles or gases. Exacerbations are common events during progression of the disease.It promotes the increase of the inflammatory process which is associated with worsening quality of life, lung function, and muscle weakness. [1, 2].

According to the American Thoracic Society and European Respiratory Society (ATS/ERS) and the American College of Chest Physicians (ACCP), a physical rehabilitation program breaks the vicious cycle of systemic manifestations, providing the patient with the maximization and maintenance of functional independence [1, 3–5].

Rehabilitation initiated during hospitalization for exacerbated COPD has great diversity as to the type (aerobic and anaerobic), intensity, and duration of exercise, as well as its influence on clinical outcomes [3, 6, 7]. Anaerobic exercise performed by lower limb muscle training during exacerbated COPD hospitalization showed improvement at the beginning of acute treatment without worsening the inflammatory process [7]. Similarly, aerobic exercise performed on a cycle ergometer during exacerbated COPD patient hospitalization was associated with improved muscle strength and postural balance; however, this study did not evaluate the effect of systemic inflammation [8].

Although scientific literature is scarce on the feasibility and effectiveness of rehabilitation programs as treatment in hospitalized exacerbated COPD patients, it is believed that rehabilitation can be initiated early during hospitalization [9, 10]. However, we did not find other simple aerobic protocols to assess the clinical benefits and any association with systemic inflammation. The hypothesis of the study is that aerobic physical exercise can be started early during hospitalization without increase in the inflammatory process and bring benefits to patients. Therefore, the objective of this study was to evaluate the influence of short-term aerobic physical exercise during hospitalization on inflammatory markers as well quality of life and physical capacity, rehospitalization, and mortality rates six months after hospital discharge in patients with exacerbated COPD.

## 2. Subjects

**Figure 1:**
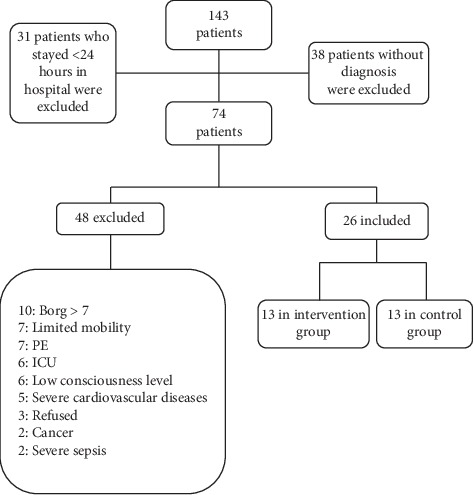
Flow diagram for patient inclusion in the study. PE, pulmonary thromboembolism; ICU, intensive care unit; Glasgow, coma scale.

We evaluated 143 hospitalized patients with a potential diagnosis of acute exacerbation of COPD (AECOPD) in the period from March 2015 to October 2016. We excluded 38 patients (26.5%) due to lack of COPD diagnosis, 31 patients who stayed less than 24 hours in hospital, and 48 (45.7%) patients for other causes: Glasgow score <15, Borg dyspnea score >7 [11], unstable heart disease, limited mobility, hemodynamic instability, and mechanical ventilation (Figure 1).

This randomized controlled clinical trial included 26 hospitalized patients; they were randomly assigned using sealed envelopes into two groups: control and intervention groups. Exacerbation was considered according to GOLD (Global Initiative for Chronic Obstructive Lung Disease) [1]. COPD was confirmed prior to hospitalization by spirometry using a bronchodilator where forced expiratory volume in one second/forced vital capacity was less than 0.70 (FEV1/FVC <0.70), and severe exacerbation was considered when hospitalization and the prescription of pharmacological treatment of patients modified in maintenance medication with the introduction of corticosteroids and/or antibiotics were needed.

This study was conducted at Botucatu Medical School Clinical Hospital, UNESP. All patients signed a free informed consent form and the study was approved by the institution ethics committee (Protocol 4027-2013). Current Controlled Trials: U1111-1166-7480.

## 3. Study Design

All patients were evaluated 24 hours after hospitalization for demographic characteristics (age, gender, occupation, education, and monthly income), smoking history (pack-year calculation and smoking status), Charlson index [12], quality of life, systemic inflammatory markers, and body composition. After 48 hours of hospitalization, all patients underwent a 6-minute walk test (6MWT) and a new spirometry test, and BODE index was calculated [13]. After 72 hours of hospitalization, patients in the intervention group underwent aerobic exercise on a treadmill for 15 minutes twice daily. Finally, a month after hospital discharge, all patients were reevaluated according to quality of life, systemic inflammatory markers, body composition, spirometry, 6MWT, and BODE index.

## 4. Methods

### 4.1. Spirometry

Spirometry was performed using a portable computerized pulmonary function system (Ferrari KoKo, Louisville, CO, USA) according to the American Thoracic Society [14]. FVC and FEV1 were measured in litres (*L*), and the FEV1/FVC was calculated. Measurements were obtained before and 20 minutes after being given a metered 400 *μ*g dose of fenoterol as a bronchodilator. FVC and FEV1 values were also expressed as a percentage of reference values [15].

### 4.2. Body Composition

Body composition was assessed by anthropometry and bioelectrical impedance (BIA 101, RJL Systems, Detroit, MI, USA). We calculated body mass index (BMI); fat-free mass (FFM) was estimated from an equation developed for patients with respiratory failure [16] from which the FFM index (FFMI = FFM(kg)/height(*m*)^2^) was calculated. Nutritional depletion was defined when FFMI <15 kg/m^2^ for women and <16 kg/m^2^ for men [17].

### 4.3. Six-Minute Walk Test

The 6MWT was performed according to the American Thoracic Society [18]. Patients with hypoxemia or who presented pulse oximetry <85% during the test were supplemented with oxygen according to medical prescription [19]. In this case, the physical therapist walked beside the patient pulling the portable cylinder trolley.

### 4.4. Quality of Life Questionnaire, Anxiety and Depression, and Intensity of Dyspnoea and Leg Fatigue

We used the validated Brazilian language and culture version of the St. George's Respiratory Questionnaire (SGRQ) [20]. Anxiety and depression level was evaluated using the HAD scale [21]. Dyspnoea intensity was evaluated using the Modified Medical Research Council (MMRC) scale, and the Borg scale for dyspnoea and leg fatigue was used during the 6MWT and aerobic exercise.

### 4.5. Systemic Inflammatory Markers

Venous blood samples were collected and centrifuged at 1000 rpm for 5 minutes. Plasma from the top of the tubes was withdrawn and centrifuged again to obtain clear plasma. Samples (220 *μ*l) were stored in a freezer at −80°C until analysis. Cytokine levels of TNF-*α* and IL-6 were performed in duplicate by commercially available immunoenzymatic (ELISA) assays (BioSource International, Inc., Ca, USA). C-reactive protein (CRP) was quantified in duplicate using ultrasound kits on Mindray BS-200 automated biochemical equipment.

### 4.6. Aerobic Exercise

Aerobic exercise was performed using a treadmill (Inbramed Master) with the patient walking for 15 minutes. Speed (m/s) was calculated from the 6MWT by dividing distance by 360 (6 minutes × 60 seconds) and the value transformed into km/h by multiplying by 3.6. The slope was increased to one point according to the Borg dyspnea scale ≤3 (moderate dyspnea) which was evaluated every five minutes during the exercise. Pulse oximeter (SpO_2_) and heart rate (HR) were monitored throughout the exercise. ECG was used to evaluate arrhythmia. The Borg scale for dyspnea and leg fatigue and respiratory rate and blood pressure were monitored at the beginning and the end of the exercise. There were two minutes of warm up and recovery using a lower speed and no inclination. Patients with hypoxemia SpO_2_ <85% during training were supplemented with O_2_ according to medical prescription.

### 4.7. Statistical Analysis

The study sample was calculated based on the difference in mean and standard deviation of the 6MWD and the total quality of life score (SGRQ) obtained in a pilot study performed in 14 patients hospitalized for AECOPD in our service. It stipulated 13 patients in each group, a statistical power of 80%, and an *α* probability of error at 5%.

All data analysis was performed using Sigmaplot 12.0 (Systat Software, Inc., San Jose, CA, USA). The data are presented in tables and continuous variables with normal distribution and are expressed as mean values with standard deviations and continuous variables with nonnormal distribution as medians and quartiles. The inflammatory values were transformed into logarithms to be normalized.

Student's *t*-test was used to compare the continuous values of two independent groups with normal distribution (e.g., control group and intervention group) and paired Student's *t*-test was used to compare the continuous values with normal distribution in the same group of patients at two different moments (e.g., variables evaluated before and after aerobic exercise). The Mann–Whitney test was used to compare the nonnormal continuous values of two independent groups, and the Wilkoxon test, to compare nonnormal values of the same group of patients at two different times. For the study between the functional and inflammatory variables, the correlations with the percentage variation of the baseline value and one month after hospital discharge were analysed using the Pearson correlation test.

Two-way ANOVA for repeated measurements was used to compare continuous variables and assess the interaction of groups and moments (initial time and one month after discharge).

## 5. Results

No differences were found between groups for general characteristics (Table 1). There was no statistical difference between proportions for disease severity (*p*=0.18). In the control group, FEV1 was moderate in 15.3% of patients, severe in 61.5%, and very severe in 15.3%. In the intervention group, it was moderate in 23%, severe in 38.4%, and very severe in 38.4%. According to the BODE index, 46.1% of control group patients presented class II, 30.7% class III, and 23% class IV. In the intervention group, 7.6% presented class II, 30.7% class III, and 61.5% class IV (*p*=0.06).

There were no statistical differences in CRP, IL-6, and TNF-*α* values before and after short-term aerobic exercise (Table 2).

The BODE index showed a statistically significant improvement one month after discharge compared to the hospitalization period without statistical difference between groups. The 6MWT did not show statistical significant interactions between groups and moments. However, we observed a statistically significant increase in the 6MWT one month after discharge compared to the hospitalization period. We did not identify statistical differences between groups, evaluation moments, and interaction according to body composition and lung function (Table 3).

There was a statistically significant improvement in BDI dyspnea score (*p*=0.003) in the intervention group between one month after and during hospitalization. The control group did not present statistical difference between these moments. Regarding MMRC, both groups showed statistically significant improvement one month after hospital discharge (the control group (0.038) and intervention group (0.005)) (Table 4).

We consistently identified a statistically significant interaction in SGRQ symptom domain; the intervention group patients presented lower respiratory symptom one month after discharge when compared to the control group. The activity and impact domain and the total score of SGRQ showed that both groups had statistically significant improvement one month after hospital discharge, but no interaction effect was observed between the factors (Table 5).

According to the systemic inflammation markers, mean CRP, IL-6, and TNF-*α* concentrations were not statistically different between groups during hospitalization and one month after hospital discharge (Table 6).

The correlations with the variation in percentage of the systemic inflammation (IL-6, TNF-*α,* and CRP) with the percentage variation of the basal value of the BDI, 6MWT variables and the domains evaluated in the quality of life by the SGRQ (symptom, activity, impact, and total). In the analysis of IL-6 variation, the variation of variable 6MWD showed a negative association with a decrease in IL-6 levels. In the analyzes of TNF-*α* and CRP, we did not identify association with the evaluated variables (Tables 7–9).

Although not statistically different, the intervention group presented two days less hospitalization time than the control group (5.0 (3.7–8.2) *vs* 7.0 (5.0–10.5) (*p*=0.28)). Eight patients (61.5%) in the control group were readmitted to hospital at least once in the six months after hospital discharge. Of these, two patients (15.3%) experienced two episodes and one three episodes. In the intervention group, four patients (30.7%) were readmitted to hospital once during the six months after hospital discharge (*p*=0.13). There were no deaths during the six months follow-up. None of the patients underwent a prior pulmonary rehabilitation.

## 6. Discussion

Our study revealed that patients with AECOPD who underwent short-term aerobic exercise during hospitalization do not worsen the inflammatory process; we observed a negative correlation of IL-6 levels in relation to 6MWD; that is, with the reduction of inflammatory levels there was an improvement in exercise capacity one month after hospital discharge. There was a significant improvement in the symptom domain evaluated by the SGRQ one month after discharge compared to patients with usual medical treatment.

We did not find any studies in the literature with the same methodology using a treadmill for aerobic exercise which evaluates systemic inflammation. We found in the literature, a study performed with patients with stable COPD compared to patients with exacerbated COPD, who underwent cycling exercise with a maximum load of 70%; mean activity time was 7 minutes, and after 30 minutes, the patients were evaluated for inflammation. High intensity exercise did not increase levels of inflammation independent of disease stability [22].

Some authors suggest that exercise session duration and intensity may be an important factor in inducing significant changes in the inflammatory process [23, 24]. Borges and Carvalho [9] showed a significant reduction in TNF-*α*, IL-6, and IL-8 levels one month after hospital discharge in their resistance exercise group, but when compared with controls, there was no significant difference. Similarly, systemic inflammation after resistance activity presented a significant reduction in CRP values similar to the control group on the eighth day of hospitalization [7].

Our results are similar to a study that evaluated the effects of aerobic exercise using an exercise bicycle during hospitalization in 58 exacerbated COPD patients. This study showed a significant improvement in equilibrium, quadriceps muscle strength, and exercise capacity in the intervention group compared with the control group on discharge from hospital; however, this study did not evaluate the effect of systemic inflammation [25]. Aerobic activity is characterized by respiratory, cardiac, and skeletal muscle strengthening. We therefore believe that performing aerobic activity during hospitalization promotes patient confidence and independence in performing daily activities and in continuing exercises after discharge, thus contributing to an active life with improved quality of life and reduced dyspnea symptoms after hospital discharge.

Endurance and aerobic exercises seem beneficial to hospitalized exacerbated COPD patients. When lower limb resistance training was performed during hospitalization, it improved physical capacity assessed by the 6MWD and muscular quadriceps strength without worsening the inflammatory process [7]. A recent study divided hospitalized COPD patients into standard medical care, one group performing only respiratory exercises and an intervention group practicing upper- and lower-limb resistance exercises. The groups in this study displayed a significant improvement in health status on discharge from hospital. However, when mobility, self-care, and usual activities were evaluated, patients performing respiratory and resistance exercises showed a significant improvement over those performing the usual medical treatment on discharge from hospital [8]. However, compared to a resistance program, aerobic training is simple and can deliver similar improvements in symptoms without the need for specific muscle training equipment.

The benefit of pulmonary rehabilitation is also related to improved quality of life and patient socialization [4, 5, 26]. Aerobic training helps improve the capacity and motor skills of already hospitalized patients and keeps them active after hospitalization, which is reflected in the improved quality of life seen one month after hospital discharge.

We did not observe a difference in mortality rate in our study after six months. However, the study did show that activity performed during the hospital phase increased mortality in the intervention group compared to the control group 12 months after hospital discharge. Patients were submitted to anaerobic training of the upper and lower limbs with three sets of 8 repetitions, neuromuscular stimulation, and aerobic exercise with daily walking with the speed determined at 85% of oxygen consumption volume estimated by the incremental shuttle test and time determined by the Borg scale of dyspnea values between 3 and 5; these patients did not present quality of life and exercise capacity benefits and mortality rates were higher in the intervention group 12 months after hospital discharge (16% in the usual care group and 25% in the intervention group). The author himself suggests an improbable association between exercise and mortality due to the positive results, such as the clinical improvement in both groups at discharge. The intervention group did not present clinical worsening after activities during the hospital period [27]. Recently, the same authors published a new study showing the benefits of rehabilitation when their intervention group was submitted to the same study exercises, and they were advised to continue walking at home with stimulation and evaluation by telephone. The results suggest that patients who underwent rehabilitation during and after hospitalization were more active and had a better quality of life [28].

Our study did not show a statistical difference between groups for the length of hospital stay. However, we did identify that our intervention group presented two days less than the control group. This has repercussions in reducing hospital costs. Compared to the literature, our value was similar to that of Borges and Carvalho [9] who reported nine days in the control group and eight days in the intervention group but was lower when compared to that of Greening et al. [27], who had on average 12 days in both groups.

Our study showed no difference between groups for readmission rate six months after discharge. Similarly, Greening et al. [27] showed that 60% of patients were readmitted to hospital without differences between groups. A Cochrane meta-analysis study evaluating the effect of pulmonary rehabilitation after exacerbation showed a reduction in risk of hospital readmission [29]. We believe there is still much to learn about the benefits of hospital-based rehabilitation. The studies we have so far show a very large variance in effects, while some authors show great benefits such as improved physical capacity, quality of life, and lower hospital readmission, and others report small improvements. For this reason, further studies are needed primarily focusing on early rehabilitation, loading, and types of exercises for AECOPD patients.

A limitation in our study is the low patient inclusion for randomization. The predominant exclusion criterion was the presentation of comorbidities which made ambulation impossible and dyspnea score high. Thus, the benefit of short-term aerobic physical training cannot be generalized for all patients with AECOPD. Literature is still scarce on hospital physical training for these patients. When evaluating training intensity and duration, we cannot affirm that increases would result in an effective clinical or quality of life improvement. We did not evaluate the activity level after discharge and we cannot assume that patients in the intervention group were more active after discharge.

The present study showed that the aerobic physical activity initiated during hospitalization in AECOPD patients does not worsen the inflammatory process. There was a negative correlation between the IL-6 marker and the 6MWD; that is, with the reduction of the inflammatory levels, there was an improvement in exercise capacity one month after hospital discharge and there was improvement of the symptom domain one month after hospital discharge in the intervention group compared to the control group.

## Figures and Tables

**Table 1:** Comparison of demographic and clinical characteristics at the baseline hospitalization according to control and intervention groups.

*N* = 26	Control group (*N* = 13)	Intervention group (*N* = 13)	*p* value
Woman (%)	69.2	61.5	0.70
Age (y)	69.3 ± 13.5	66.8 ± 9.49	0.58
Smoking (pack-years)	50.0 (31.8–92.0)	46.0 (40.0–68.0)	0.87
LBMI (kg/m^2^)	16.0 (14.8–17.0)	15.7 (14.6–17.4)	0.43
BMI (kg/m^2^)	23.3 ± 4.69	25.4 ± 5.29	0.41
SGRQ			
Symptom (%)	72.8 ± 4.7	79.2 ± 4.5	0.52
Activity (%)	85.8 ± 3.9	91.5 ± 3.7	0.26
Impact (%)	46.5 ± 5.5	60.6 ± 5.3	0.44
Total (%)	62.7 ± 4.5	73.0 ± 4.3	0.53
Anxiety, score	7.4 ± 5.0	8.9 ± 6.9	0.89
Depression, score	5.3 ± 4.4	3.1 ± 3.1	0.04
CRP (mg/dl)	27.2 (7.30–50.52)	8.35 (7.31–29.4)	0.34
TNF-*α* (mg/dl)	1.57 (1.17–1.77)	1.60 (1.07–1.69)	0.64
IL-6 (mg/dl)	3.68 (1.60–7.10)	2.01 (1.65–5.46)	0.56
FEV_1_ (*L*)	0.89 ± 0.32	0.75 ± 0.30	0.14
FVC (%)	60.1 ± 11.8	55.7 ± 17.0	0.55
FVC (*L*)	1.82 ± 0.60	1.63 ± 0.52	0.28
FEV_1_/FVC (*L* pH)	0.49 (0.47–0.56)	0.45 (0.38–0.48)	0.05
PaO_2_ (mmhg)	7.4 ± 0.0	7.4 ± 0.0	0.37
PaCO_2_ (mmhg)	56.1 ± 12.7	54.4 ± 14.7	0.83
SaO_2_ (%)	48.4 ± 5.2	47.7 ± 10.9	0.89
BODE	87.4 ± 4.8	84.8 ± 9.6	0.53
6MWD (*m*)	5.30 ± 1.88	6.53 ± 1.68	0.08
	219.0 ± 136.8	190.7 ± 94.3	0.34

Values are expressed as mean ± standard deviation or median (quartile 1–quartile 3). LBMI, lean body mass index; BM,: body mass index; SGRQ, Saint George's Respiratory Questionnaire; CRP, C-reactive protein; TNF-*α*, tumor necrosis factor alpha; IL-6, interleukin 6; FEV1, forced expiratory volume in one second; FVC, forced vital capacity; PaO2, arterial oxygen pressure; pH, hydrogenation potential; PaCo2, arterial pressure of carbon dioxide; SaO2, arterial oxygen saturation; BODE, body mass index, airway obstruction, dyspnea, and exercise capacity; 6MWT, six-minute walk test; *p* < 0.05, comparisons evaluated by the *t* test and Mann–Whitney test.

**Table 2:** Evaluation of systemic inflammatory values before and after short-term aerobic exercise.

*N* = 13	Start of the test	End of the test	*p* value
TNF-*α* (mg/dl)	1.19 (0.99–1.71)	1.21 (0.77–1.53)	0.58
IL-6 (mg/dl)	2.41 (2.02–0.58)	2.66 (1.69–0.48)	0.21
CRP (mg/dl)	3.88 (2.26–8.04)	4.07 (2.65–13.3)	0.56

Values expressed as median (quartile 1–quartile 3). TNF-*α*, tumor necrosis factor alpha; IL-6, interleukin 6; CRP, C-reactive protein. *p* < 0.05, comparisons evaluated by the Wilkoxon test.

**Table 3:** Comparison of evaluation moments and groups in relation to body composition, lung function, 6MWT, and BODE index.

*N* = 26	Control group (*N* = 13)		Intervention group (*N* = 13)		*p* time	*p* group	*p* TxG
	Hospitalization	After one month	Hospitalization	After one month			

BMI	23.3 ± 4.4	24.2 ± 6.1	25.4 ± 4.8	26.5 ± 5.09	0.89	0.18	0.33
LBMI	16.0 ± 3.6	15.6 ± 6.8	15.7 ± 2.1	17.5 ± 2.1	0.36	0.45	0.38
FEV1 (%)	39.0 ± 9.6	40.4 ± 15.0	32.6 ± 15.2	40.9 ± 14.1	0.05	0.51	0.45
FEV1 (*L*)	0.89 ± 0.29	1.02 ± 0.41	0.74 ± 0.32	0.91 ± 0.28	0.12	0.75	0.4
FVC (%)	60.1 ± 12.2	68.7 ± 18.2	55.7 ± 18.8	63.5 ± 18.5	0.09	0.89	0.55
FVC (*L*)	1.82 ± 0.64	2.05 ± 0.78	1.63 ± 0.57	1.82 ± 0.51	0.23	0.87	0.49
FEV1/FVC (*L*)	0.45 ± 0.09	0.50 ± 0.09	0.45 ± 0.09	0.50 ± 0.10	0.05	0.95	0.95
BODE	5.6 ± 1.9	5.0 ± 2.3	6.3 ± 1.7	4.4 ± 2.0	**0.002**	0.85	0.24
6MWT	219.0 ± 38.4	269.5 ± 28.1	190.7 ± 40.3	277.4 ± 29.5	**0.02**	0.97	0.26

Values are expressed as mean ± standard deviation. LBMI, lean body mass index; BMI, body mass index; FEV1, forced expiratory volume in one second; FVC, forced vital capacity; 6MWT, six-minute walk test; BODE, body mass index, airway obstruction, dyspnea, and exercise capacity; *p* < 0.05, comparison evaluated by the two-way ANOVA test with repeated measures.

**Table 4:** Comparison of evaluation moments and groups in relation to BDI, MMRC, and anxiety and depression.

*N* = 26	Control group (*N* = 13)		Intervention group (*N* = 13)		*p* time	*p* group	*p* TxG
	Hospitalization	After one month	Hospitalization	After one month			
BDI	4.1 ± 3.0	5.6 ± 2.4	2.4 ± 1.6	5.0 ± 1.4	**0.008**	0.39	0.15
MMRC	2.7 ± 0.9	2.0 ± 1.0	3.1 ± 0.4	2.2 ± 0.6	**0.001**	0.24	0.67
Anxiety	7.4 ± 5.0	4.5 ± 5.4	8.9 ± 6.9	4.0 ± 4.9	**0.04**	0.80	0.60
Depression	5.3 ± 4.4	3.3 ± 3.9	3.1 ± 3.1	2.3 ± 2.2	0.22	0.17	0.63

Values are expressed as mean ± standard deviation. BDI, basal dyspnea index; P TxG, *p* value of the interaction between time and group. *p* < 0.05, comparison assessed by two-way ANOVA repeated measurements analysis.

**Table 5:** Comparison of evaluation moments and groups in relation to SGRQ and dyspnea score.

*N* = 26	Control group (*N* = 13)		Intervention group (*N* = 13)		*p* time	*p* group	*p* TxG
	Hospitalization	After one month	Hospitalization	After one month			
Activity	85.8 ± 3.9	63.0 ± 6.4	91.5 ± 3.7	66.3 ± 6.1	**<0.001**	0.53	0.84
Impact	46.5 ± 5.5	31.7 ± 5.2	60.6 ± 5.3	31.8 ± 5.2	**0.02**	0.36	0.20
Symptom	72.8 ± 4.7	63.8 ± 4.7	79.2 ± 4.5	50.5 ± 4.5	**0.001**	0.38	**0.04**
Total	62.7 ± 4.5	47.9 ± 5.0	73.0 ± 4.3	44.5 ± 4.8	**<0.001**	0.57	0.21

Values are expressed as mean ± standard deviation. SGRQ, Saint George's Respiratory Questionnaire*p* < 0.05, comparison evaluated by the two-way ANOVA test with repeated measures.

**Table 6:** Comparison of evaluation moments and groups in relation to systemic inflammation.

*N* = 26	Control group (*N* = 13)		Intervention group (*N* = 13)		*p* time	*p* group	*p* TxG
	Hospitalization	After one month	Hospitalization	After one month			
Log CRP (mg/dl)	2.91 ± 0.64	2.15 ± 0.56	2.65 ± 0.54	2.48 ± 0.37	0.37	0.95	0.44
logTNF-α (mg/dl)	0.61 ± 0.14	0.66 ± 0.29	0.67 ± 0.22	0.32 ± 0.10	0.60	0.58	0.07
Log IL-6 (mg/dl)	1.30 ± 0.61	1.10 ± 0.12	1.40 ± 0.52	0.52 ± 0.74	0.30	0.99	0.34

Values are expressed as mean ± standard deviation. Log, transformed into logarithms; CRP, C-reactive protein. TNF-α, tumor necrosis factor alpha; IL-6, interleukin 6; P TxG, *p* value of the interaction between time and group. *p* < 0.05, comparison evaluated by the two-way ANOVA test with repeated measures.

**Table 7:** Correlation of IL-6 levels with the independent variables.

Variable dependent	Variables	Correlation	*p* value
	BDI	0.50	0.07
	6MWD	−0.66	**0.01**
ΔIL-6 (%)	Symptom	−0.10	0.73
	Activity	−0.41	0.16
	Impact	−0.02	0.94
	Total	−0.15	0.60

IL-6, interleukin-6; 6MWD, six-minute walk test; BDI, basal dyspnea index. *p* < 0.05, comparison evaluated by the Pearson correlation test.

**Table 8:** Correlation of TNF-*α* levels with the independent variables.

Variable dependent	Variables	Correlation	*p* value
	BDI	−0.32	0.20
	6MWD	0.09	0.70
ΔTNF-*α* (%)	Symptom	0.23	0.36
	Activity	0.47	0.05
	Impact	0.27	0.29
	Total	0.35	0.16

TNF-α, tumor necrosis factor alpha; 6MWD, six-minute walk test; BDI, basal dyspnea index. *p* < 0.05, comparison evaluated by the Pearson correlation test.

**Table 9:** Correlation of PCR levels with the independent variables.

Variable dependent	Variables	Correlation	*p* value
	BDI	0.39	0.11
	6MWD	0.33	0.18
ΔCRP (%)	Symptom	−0.20	0.42
	Activity	−0.05	0.82
	Impact	−0.16	0.51
	Total	−0.16	0.53

CRP, C-reactive protein; 6MWD, six-minute walk test; BDI, basal dyspnea index. *p* < 0.05, comparison evaluated by the Pearson correlation test.

## Data Availability

The data used to support the findings of this study are available from the corresponding author upon request.
